# Increase of OH radical yields due to the decomposition of hydrogen peroxide by gold nanoparticles under X-ray irradiation†

**DOI:** 10.1039/d4ra00208c

**Published:** 2024-03-20

**Authors:** Yu Okazaki, Tamon Kusumoto, Stephane Roux, Ryoichi Hirayama, Michel Fromm, Rana Bazzi, Satoshi Kodaira, Jun Kataoka

**Affiliations:** a Graduate School of Advanced Science and Engineering, Waseda University 3-4-1 Okubo, Shinjuku-ku Tokyo 169-8555 Japan; b National Institutes for Quantum Science and Technology (QST) 4-9-1 Anagawa, Inage-ku Chiba 263-8555 Japan kusumoto.tamon@qst.go.jp; c UMR CNRS 6249 Chrono-Environnement, Université de Franche-Comté F-25030 Besançon Cedex France

## Abstract

We elucidate the decomposition mechanism of hydrogen peroxide, which is formed by water radiolysis, by gold nanoparticles (GNPs) under X-ray irradiation. The variations in yields of hydrogen peroxide generated in the presence of GNPs are evaluated using the Ghormley technique. The increase of yields of OH radicals has been quantified using Ampliflu® Red solutions. Almost all hydrogen peroxide generated by irradiation of <25 Gy is decomposed by GNPs, while the yield of OH radicals increases by 1.6 times. The amount of OH radicals thus obtained is almost equivalent to that of the decomposed hydrogen peroxide. The decomposition of hydrogen peroxide is an essential reaction to produce additional OH radicals efficiently in the vicinity of GNPs.

## Abstract 2

We elucidate the decomposition mechanism of hydrogen peroxide, which is formed by water radiolysis, by gold nanoparticles (GNPs) under X-ray irradiation.

## Graphical abstract

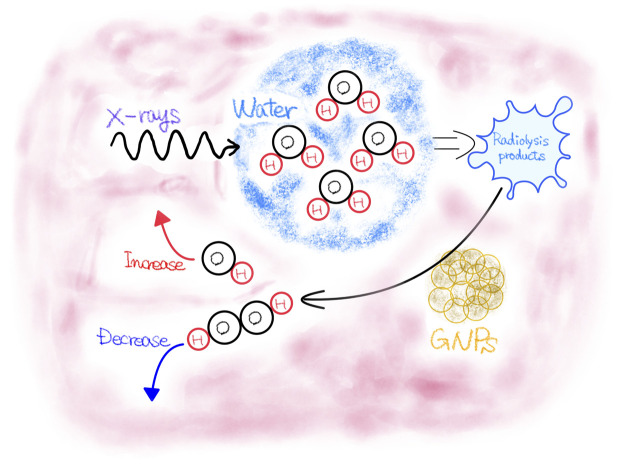

## Introduction

Ionizing radiations induce ionization and excitation of water molecules by transferring their energies. Such processes lead to the production of water radiolysis products.^1,2^ Yields of water radiolysis products (*e.g.*, OH radicals, hydrogen peroxide and hydrated electrons) in diluted solutions have been evaluated in various studies (*e.g.*, ref. 3–6). Gold nanoparticles (GNPs) with the function of inducing reactive oxygen species (ROS) overexpression may have potential as radiation sensitizers.^7^ The effectiveness of GNPs would vary based on the nanoparticles' size, types, and concentration, radiation types, incident beam energy, synthesis method, *etc.*^8^ Therefore, further investigations are necessary for explaining the mechanism of GNPs as radiation sensitizers.

Fundamental studies aiming at the elucidation of the radio-sensitization mechanisms of GNPs have been actively undertaken.^9,10^ From a radiation chemistry viewpoint, the enhancement mechanism would be related to the increase of yields of OH radicals,^11,12^ which efficiently react with DNA and proteins.^13^ Therefore, changes in yields of OH radicals are one of the key phenomena to elucidate the sensitization mechanism. In this regard, it was reported that hydrogen peroxides could be activated by GNPs to OH radicals.^8^ Thus, changes in yields of OH radicals and hydrogen peroxide by GNPs will lead us to understand the mechanism(s). In the present study, we focused on changes in yields of OH radicals and hydrogen peroxide with and without GNPs to unveil a part of the mechanisms of the radio-sensitization mechanism.

## Experiments

### Synthesis of GNPs

**Fig. 1:**
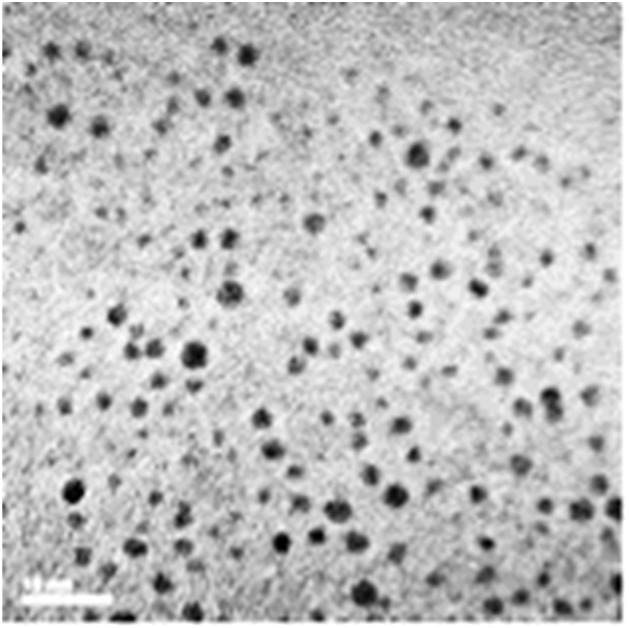
A transmission electron micrograph image of Au@DTDTPA nanoparticles (scale bar: 10 nm).

Since the chelator-coated GNPs (Au@DTDTPA) exhibit a high potential for image-guided radiotherapy,^14^ they were chosen for evaluating their potential to amplify the production of OH radicals, which could explain their efficacy for increasing the life span of animals treated by radiotherapy after intravenous injection of Au@DTDTPA nanoparticles. The gold salt was reduced by sodium borohydride in presence of Au@DTDTPA down to a core size between 2 and 3 nm and a hydrodynamic diameter ranging from 7 and 10 nm (Fig. 1).^15,16^ The detail of the synthesize protocol of Au@DTDTPA was described in a supplemental material. The concentration of this Au@DTDTPA in this study was 2.5 μg mL^−1^ in the solutions for evaluating yields of water radiolysis products.

### Chemicals

Hydrogen peroxide is mainly decomposed by hydrated electrons. To minimize the influence, we prepared 1 mM NaNO_3_ (Fujifilm/Wako Pure Chemical Industries Ltd.) solutions from ultra-pure water using a Millipore Milli-Q system. Here, NaNO_3_ acts as a scavenger of hydrated electrons with the rate constant *k* = 9.7 × 10^9^ M^−1^ s^−1^. The product of the *k* and the concentration of the scavenger indicates the scavenging capacity, *S*.^17^ The inverse of the scavenging capacity corresponds to the scavenging time scale, *τ*. Therefore, using 1 mM NaNO_3_ solution, we can evaluate primary yields of hydrogen peroxide.^17^

We used Ampliflu® Red (*N*-acetyl-3,7-dihydroxyphenoxazine) probes (Sigma Aldrich) for OH radical measurements, which enables highly sensitive detection without any separation step of molecular products, *via* chromatography for example.^12,18^ Ampliflu® Red is known as a scavenger of OH radicals with *k* = 5.0 × 10^9^ M^−1^ s^−1^.^18^ The concentration of the prepared solution was 50 μM, thereby we evaluated primary yields of OH radicals.

### X-ray irradiations

The prepared solutions contained in 2 mL Eppendorf Tubes (WATOSON BIO LAB.) were irradiated to X-rays using an X-ray generator (PANTAK HF320-S, Shimadzu) operating at 200 kVp and 20 mA with a 0.5 mm aluminum and 0.5 mm copper filters. Continuous X-rays with an effective energy of 83 keV were used, meaning that energies of secondary electrons are widely distributed. Therefore, it is not possible to focus on the contribution of Auger electrons seen under monochromatic X-rays.^19^

### Analysis

The concentration of hydrogen peroxide formed by irradiation was quantified using Ghormley technique.^20^ We measured the absorbance at 352 nm with the molar extinction coefficient of 23 800 M^−1^ cm^−1^ using a UV-Visible spectrophotometer (UV1900S, Shimadzu).

OH radicals formed by water radiolysis react with a benzene ring in Ampliflu® Red, and then a highly fluorescent product, resorufin (Sigma Aldrich), is generated.^12^ Namely, yields of resorufin are proportional to those of OH radicals. We measured the fluorescence intensity of emissions at 582 nm from resorufin in the solutions by excitation at 532 nm using a fluorescence spectrophotometer (RF-6000, Shimadzu).

## Considerable roles played by GNPs to water radiolysis

**Fig. 2:**
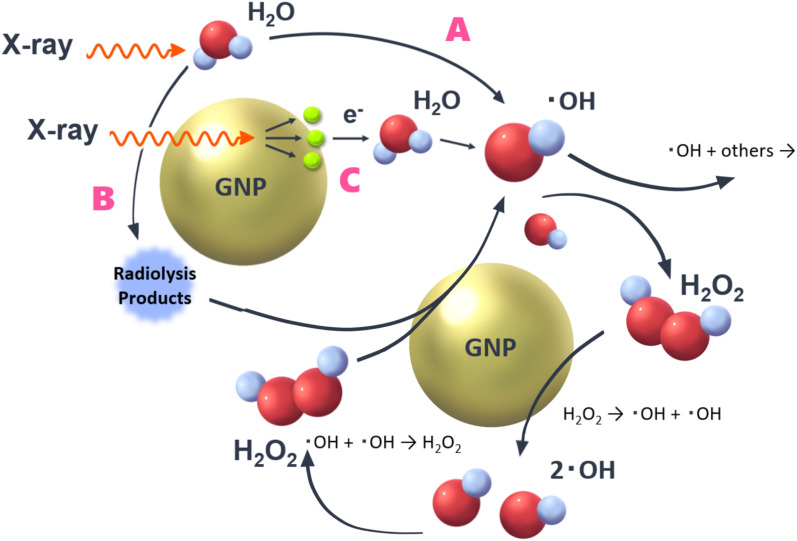
Three pathways to form OH radicals by water radiolysis.^8,11^ GNPs could contribute to rise OH radical yields *via* pathways B and C.

In a previous study, the following important pathways related to OH radical production were proposed^8,11^ (Fig. 2),

● Pathway A: direct reactions of water molecules with X-rays. This is an ordinary pathway of water radiolysis. Not only OH radicals but also hydrogen peroxide (H_2_O_2_) and hydrated electrons are generated.

● Pathway B: secondary reactions between ROS and GNPs.

● Pathway C: contribution of low-energy secondary electrons, which are generated by reactions of X-rays with GNPs.

As briefly mentioned above, pathway A is an ordinal one of water radiolysis, thereby yields of water radiolysis species (*e.g.*, OH radicals, hydrogen peroxide and hydrated electrons) are not affected in this pathway by the presence of GNPs. In pathway C, low-energy secondary electrons (*e.g.*, Auger electrons) launched from GNPs contribute to the decomposition of water molecules.^21^ It has been reported that the irradiation of particles with higher linear energy transfer (LET) results in lower yields of OH radicals.^22^ So, the pathway C would not contribute the increase of yields of OH radicals. However, the increase of OH radical yields observed by adding GNPs in previous studies.^11,12^ This would support pathway B, for example, hydrogen peroxide, which is formed by water radiolysis (˙OH + ˙OH → H_2_O_2_) is decomposed by GNPs, leading to OH radicals and/or OH^−^. Some OH radicals react with each other, resulting in hydrogen peroxide formation again. So, the reaction shown in pathway B seems to keep looping. However, this loop reaction will end within a finite time by reactions of newly formed OH radicals with other surrounding water radiolysis products such as hydrated electrons. Although some other reactive species can react with GNPs, an essential one related to the mechanism is supposed to be hydrogen peroxide because OH radicals are formed additionally by its decomposition.^23^

The relationship between the yields of hydrogen peroxide and OH radical decomposition has not yet been quantitatively evaluated for GNP exposed in aqueous solution to ionizing radiation. This ratio was only determined experimentally by deduction, using an ROS scavenging method.^8^ As is well known, OH radicals efficiently react with DNA and proteins, resulting in DNA single strand breaks due to the indirect action.^13^ Hydrogen peroxide also triggers DNA strand breaks without impairing cell survival. A linear-quadratic increase of DNA double strand breaks was seen with increasing the concentration of hydrogen peroxide, so that Dahm-Daphi *et al.* interpreted that these damages would be due to a single or pairwise action of OH radicals.^24^ Thus, it is very important to evaluate the relation between OH radicals and hydrogen peroxide in the presence of GNPs. Note here that this relation may lead the clarification of roles played by enzymes, for instance catalase and peroxidases, to the decomposition of hydrogen peroxide. In this study, we evaluate the increase of OH radical yields associating with changes in yields of hydrogen peroxide in diluted solutions as a first fundamental step toward the elucidation of the radio-sensitization mechanism of GNPs with high scavenging condition (*e.g.*, in living cells).

## Results and discussion

### Yields of hydrogen peroxide

**Fig. 3:**
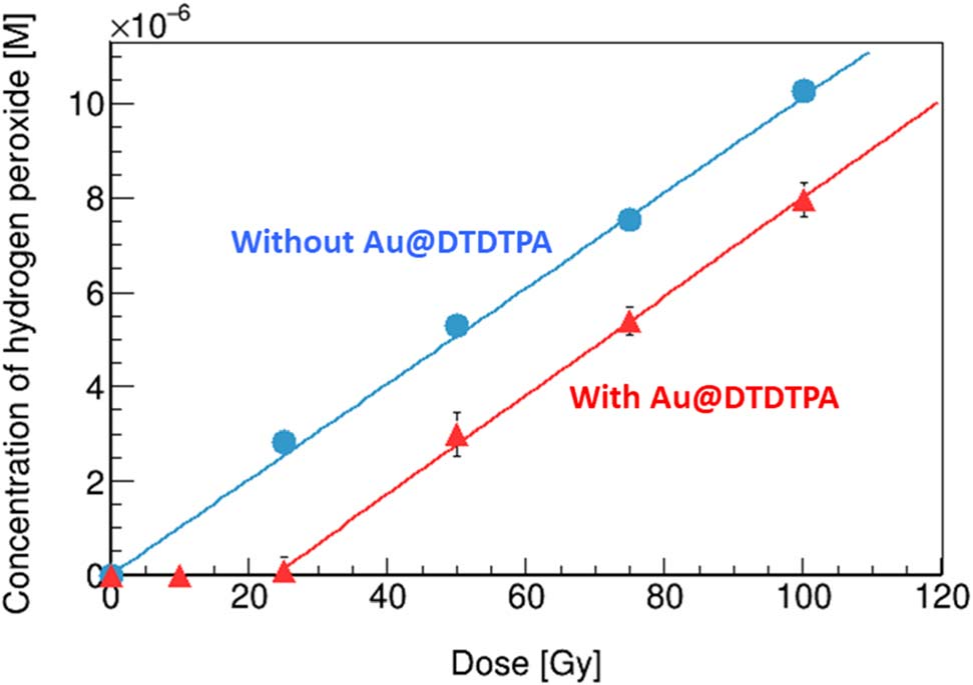
Variations of hydrogen peroxide concentrations in water with/without Au@DTDTPA as a function of X-ray absorbed dose. Error bars correspond to the standard deviations of prepared solutions (*N* ≥ 3).

As shown in Fig. 3, the concentration of hydrogen peroxide increases linearly with the rise in absorbed dose, in the solution without Au@DTDTPA (circles). From the slope of the fitting line, radiation chemical yields (G value), expressed as the number of entities formed (or lost) per unit energy (traditionally 100 eV), were evaluated. The G value of hydrogen peroxide in water under X-ray irradiation was 1.0 sp/100 eV (= species/100 eV), which is slightly higher than that of previously reported value by gamma rays from Co-60 of 0.7 sp/100 eV.^17^ The effective energy of the present X-ray beam (83 keV) is considerably lower than that of gamma rays from Co-60 (1.17 and 1.33 MeV). Overall, these observations are in good agreement with the conclusions drawn by Fulford and co-workers,^25^ concerning the yield of OH radicals escaping radiation tracks (*i.e.*, which escape intra-track recombination) as a function of the initial energy of Low-LET radiations (X-rays and gamma-rays) (see a supplemental material). As the photon energy decreases from ∼1 MeV to 83 keV, the OH radical yield decreases in line with an increased ionization density of the radiation and hence an increasing probability of radical recombination, leading the increase of hydrogen peroxide formation.^26^ In the solution with Au@DTDTPA (triangles), no production of hydrogen peroxide was observed up to 25 Gy, then it increases monotonically with increase of the absorbed dose. It has been observed that hydrogen peroxide is decomposed on the surface of GNPs.^27^ In the present case, all hydrogen peroxide formed is decomposed by Au@DTDTPA below 25 Gy. At 25 Gy, the concentration of hydrogen peroxide formed is 2.2 μM (see circles in Fig. 3). Thus, the decomposing capacity of Au@DTDTPA with 2.5 μg mL^−1^ for hydrogen peroxide would be considered as 2.2 μM under X-ray irradiations. Above 25 Gy, the amount of hydrogen peroxide produced in water radiolysis exceeds the ability of the Au@DTDTPA to decompose them. Namely, above 25 Gy, the decomposition of hydrogen peroxide and the associated generation of OH radicals, illustrated as pathway C in Fig. 2, could be under an equilibrium condition. When solutions were irradiated, the slope with Au@DTDTPA above 25 Gy is almost equivalent to that without Au@DTDTPA. This finding indicates that Au@DTDTPA decomposed almost hydrogen peroxide formed by water radiolysis below 25 Gy.

### Yields of OH radicals

**Fig. 4:**
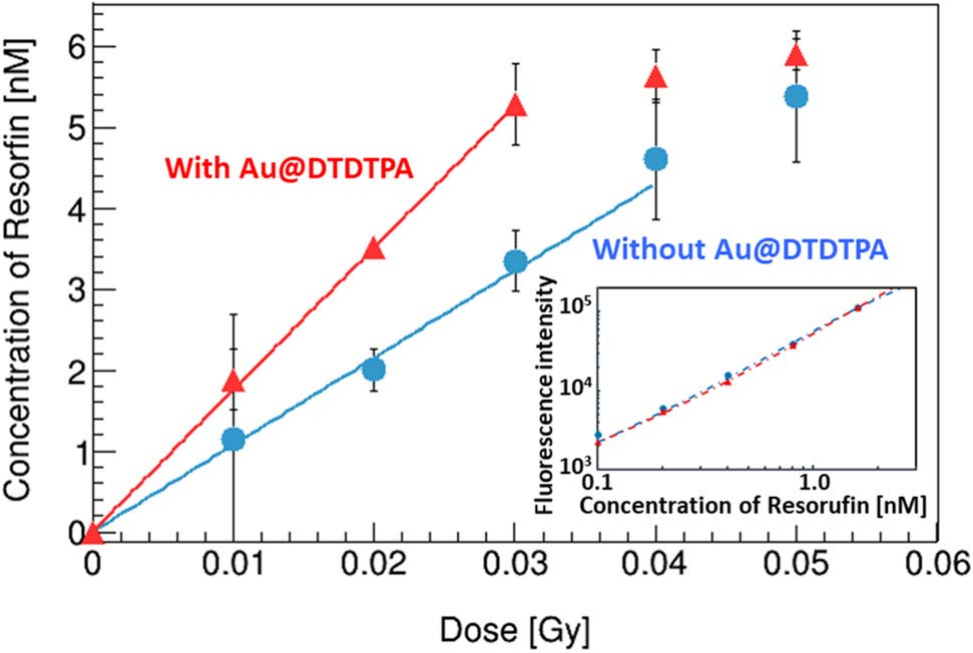
Variations of the resorufin concentrations in water with/without Au@DTDTPA as a function of X-ray absorbed dose. The inset is calibration data of the resorufin concentrations.

A calibration curve of resorufin was made (inset of Fig. 4). The fluorescence intensity of resorufin s with Au@DTDTPA (triangles) is almost equivalent to that without Au@DTDTPA (circles). This result indicates that the resorufin formed is not affected by adding Au@DTDTPA.

Fig. 4 represents the increase of the resorufin concentration as a function of X-ray dose. When Au@DTDTPA are added in the solution, it rapidly increased up to 0.03 Gy (triangles) compared to that without Au@DTDTPA (circles) and then saturated. The G values of the resorufin below 0.03 Gy were 1.2 and 2.0 sp/100 eV without and with Au@DTDTPA, respectively.

### Quantitative discussion for the increase of OH radicals

The G value of OH radicals in water for 83 keV X-rays was reported as 2.6 sp/100 eV by others.^26,28,29^ The ratio of measured G value of resorufin without Au@DTDTPA, 1.2 sp/100 eV, to reported G value of OH radicals in water, 2.6 sp/100 eV, is about 0.46. This ratio is significantly higher than those of other probes, such as coumarin-3-carboxylic acid and terephthalic acid of 0.05 (ref. 22) and 0.32,^30^ respectively. The G value of resorufin with Au@DTDTPA was 1.6 times higher than that without Au@DTDTPA. The present result agrees with previous observations: 2.2 times with irradiation of 20 keV photons,^11,31^ 2.2 times with irradiation of alpha particles.^12^ Thus, the G value of OH radicals with Au@DTDTPA was determined as 4.2 sp/100 eV.

In the examined region of OH radical yields, almost all hydrogen peroxide formed by irradiation was decomposed by Au@DTDTPA. Thus, G value of OH radical formed additionally was 2.0 sp/100 eV (namely, twice of G value of hydrogen peroxide). The G value of OH radicals measured by Ampliflu® Red increased by 1.6 sp/100 eV by adding Au@DTDTPA. This value is about 20% lower than the G value of OH radicals formed additionally from hydrogen peroxides, which suggests that some OH radicals additionally formed react with other water radiolysis products (*e.g.*, ˙OH + e_aq_^−^ → OH^−^, *k* = 2.95 × 10^10^ M^−1^ s^−1^; ˙OH + ˙H → H_2_O, *k* = 7.0 × 10^9^ M^−1^ s^−1^). Furthermore, the generation of OH^−^ due to the decomposition of hydrogen peroxide (H_2_O_2_ → ˙OH + OH^−^) would be influenced to the ratio.

The present study experimentally demonstrates the production of additional OH radicals due to the decomposition of hydrogen peroxide at Au@DTDTPA surface. It has been performed using diluted solutions, which enable us to evaluate primary yields of water radiolysis. In living cells, they have high scavenging capacity, so that lifetime of water radiolysis products is considered as a few nanoseconds. Both single and multiply DNA damages are induced by incoming particles, when radical mobility is limited by the presence of radical scavengers (*e.g.*, in living cells).^32^ It was reported that biological consequences would be related to the detailed spatial and temporal nature of radiation initial features.^33^ Thus, to unveil the radio-sensitization mechanisms of GNPs properly and accurately, ones must focus on the initial damage process in a few nanoseconds scale in further steps.

## Conclusions

We investigated the role played by Au@DTDTPA to decompose hydrogen peroxide, leading the additional formation of OH radicals, in diluted solutions. The G value of hydrogen peroxide formed was 1.0 sp/100 eV. Almost hydrogen peroxide formed by X-ray irradiation was decomposed by adding Au@DTDTPA below 25 Gy. As a result, the G value of OH radicals in water with Au@DTDTPA increased by 1.6 times higher than that without Au@DTDTPA. This value was reasonable with previously obtained ones under X-rays and alpha irradiations.

## Conflicts of interest

There are no conflicts to declare.

## Supplementary Material

RA-014-D4RA00208C-s001
