# Supplementary material for: Increase of OH radical yields due to the decomposition of hydrogen peroxide by gold nanoparticles under X-ray irradiation
Source: RSC Adv. 2024 Mar 20;14(14):9509–13. doi: 10.1039/d4ra00208c (PMC10953845; doi:10.1039/d4ra00208c)
Supplement: RA-014-D4RA00208C-s001 [file RA-014-D4RA00208C-s001.pdf]

Electronic Supplementary Material (ESI) for RSC Advances.
This journal is © The Royal Society of Chemistry 2024

Supplimental material

Yu Okazaki[1], Tamon Kusumoto[2*], Stephane Roux[3], Ryoichi Hirayama[2], Michel Fromm[3], Rana Bazzi[3], Satoshi Kodaira[2], Jun Kataoka[1],

[1] *Graduate School of Advanced Science and Engineering, Waseda University, 3-4-1 Okubo, Shinjuku-ku, Tokyo 169-8555, Japan*

[2] *National Institutes for Quantum Science and Technology (QST), 4-9-1 Anagawa, Inage-ku, Chiba 263-8555, Japan*

[3] *UMR CNRS 6249 Chrono-environnement, Université de Franche-Comté, F-25030 Besançon Cedex, France*

Synthesis of Au@DTDTPA nanoparticles[S1,S2]

Hainfeld and his colleagues demonstrated that ultrasmall gold nanoparticles permits to increase significantly both the contrast of X-ray imaging and the efficiency of the radiotherapy[S3]. This study is probably at the origin of the intense research activity devoted to the development of radioenhancing nanoparticles for image-guided radiotherapy. Due to its low sensitivity and its ionizing character, X-ray imaging is probably less suited for image-guided radiotherapy than magnetic resonance imaging (MRI). In this context, we developed the synthesis of gold nanoparticles coated with linear (DTDTPA) or macrocyclic (TADOTAGA) polyaminocarboxylate chelators by adapting Brust's protocol. This protocol is well suited for providing ultrasmall gold nanoparticles with a high colloidal stability in aqueous solution. The reduction of gold salt by sodium borohydride in presence of chelators yields chelator-coated gold nanoparticles with a core size (see Figure 2 in the manuscript) between 2 and 3 nm and a hydrodynamic diameter ranging from 7 and 10 nm[S1,S2].

The chelator-coated gold nanoparticles are therefore composed of two different parts which play important and complementary roles. The organic shell confers to the nanoparticles a high colloidal stability in biological media, ensures a long-term immobilization of Gd for monitoring their biodistribution by MRI and also the immobilization of radioisotopes for nuclear imaging while the radioenhancing effect and the CT imaging modality stem from the gold core[S4-S6]. Finally, the imaging experiments by MRI and nuclear imaging highlight the safe behavior of these nanoparticles since they are only present in 9 L gliosarcoma and in the organs involved in the renal excretion after intravenous injection. Such a preferential accumulation was exploited for increasing the lifespan of rats bearing a brain tumor in the right hemisphere [S4]. The treatment of the rats by radiotherapy after intravenous injection of gadolinium chelate-coated gold nanoparticles induced a longer survival in comparison to the non-treated rats (with an increase of lifespan (ILS) of 473%) and to the rats treated only by radiotherapy (with an ILS of 78%).

Since these chelator-coated gold nanoparticles (Au@DTDTPA) exhibit a high potential for image-guided radiotherapy, they were chosen for evaluating their potential to amplify the

production of OH radicals which could explain their efficacy for increasing the lifespan of animals treated by radiotherapy after intravenous injection of Au@DTDTPA nanoparticles.

The synthesis, adapted from a protocol described by Brust *et al.*, consists in reducing $HAuCl_4,3H_2O$ by $NaBH_4$ in presence of thiols (stabilizers) which, by adsorption on growing particles, ensure the control of the size and the stability of the colloid[S7].

For a typical preparation of gold particles, 200 mg (51×10^{-5} mol) of $HAuCl_4,3H_2O$, dissolved in 60 mL methanol were placed in a 250 mL round bottom flask. 256 mg (50×10^{-5} mol) of DTDTPA in 40 mL of water and 2 mL of acetic acid were added to the gold salt solution under stirring. The mixture turned from yellow to orange. After 5 minutes, 185 mg (489×10^{-5} mol) of $NaBH_4$ dissolved in 14 mL water were added to the orange mixture under vigorous stirring at room temperature. At the beginning of the $NaBH_4$ addition, the solution became first dark brown then a black floculate appeared.

The vigorous stirring was maintained for 1h before adding 5 mL of 1 M aqueous hydrochloric acid solution. After the partial removal of the solvent under reduced pressure and at maximum 40°C, the precipitate was retained on a polymer membrane (pore diameter 0.22 μm), washed thoroughly and successively with 0.01 M HCl, water and diethyl ether. The resulting black powder (Au@DTDTPA) was dried and dispersed in 10 mL of 0.01 M NaOH solution (with a fixed concentration ranging from 1 to 10 g Au/L).

Energy dependence of OH radical yields

As is well known, under ionizing radiations, tacks are formed by separate clusters of reactive species (might be spherical in shape). The separate cluster is generally known as “spur”[S8]. The spurs form dense continuous columns with increasing linear energy transfer (LET). Concequently, the tracks become cylindrical in shape, resulting in the reduction of primary yields (~ 100 ns) of reactive species (e.g., OH radicals and hydrated electrons) with increasing LET. Namely, primary yields of OH radicals decrease with decreasing energy of photons. Here, we summarize the energy dependence of primary yields of OH radicals under photon irradiations (Table S1). Fuloford and co-workers estimated primary yields of OH radicals from yields of single strand breaks of plasmid DNA[S9]. Furtheremore, we presented primary yields under 662 keV photons evaluated by a Monte Carlo simulation[S10].

Table S1. Primary yields of OH radicals with photon energy.

Primary yields (sp/100 eV)	Type of electromagnetic radiation	Reference
2.9	gamma rays (Co-60)	
2.4	X-rays (90 keV)	
2.1	X-rays (23 keV)	
0.9	X-rays (4.55 keV)	Fulford et at., 1999 [S9].
0.7	X-rays (1.49 keV)	
0.8	X-rays (0.96 keV)	
2.5	X-rays (0.28 keV)	
2.7	gamma rays (Cs-137)	Meesungnoen et al., 2001 [S10].

References

S1. P.-J. Debouttière, S. Roux, F. Vocanson, C. Billotey, O. Beuf, A. Favre-Réguillon, Y. Lin, S. Pellet-Rostaing, R. Lamartine, P. Perriat and O. Tillement, *Advanced Functional Materials*, 2006, **16**, 2330-2339.

S2. C. Alric, J. Taleb, G. Le Duc, C. Mandon, C. Billotey, A. Le Meur-Herland, T. Brochard, F. Vocanson, M. Janier, P. Perriat, S. Roux and Tillement, *J. Am. Chem. Soc.*, 2008, **130**, 5908-5915.

S3. J.F. Hainfeld, D.N. Slatkin, H.M. Smilowitz, *Phys. Med. Biol*. 2004, **49(18)**, N309-315.s

S4. C. Alric, J. Taleb, G. Le Duc, C. Mandon, C. Billotey, A. Le Meur-Herland, T. Brochard, F. Vocanson, M. Janier, P. Perriat, S. Roux and Tillement, *J. Am. Chem. Soc.*, 2008, **130**, 5908-5915.

S5. C. Alric, I. Miladi, D. Krysa, J. Taleb, F. Lux, R. Bazzi, C. Billotey, M. Janier, P. Perriat, S. Roux and O. Tillement, *Nanoscale*, 2013, **5**, 5930-5939.

S6. I. Miladi, C. Alric, S. Dufort, P. Mowat, A. Dutour, C. Mandon, G. Laurent, E. Bräuer-Krisch, N. Herath, J.-L. Coll, M. Dutreix, F. Lux, R. Bazzi, C. Billotey, M. Janier, P. Perriat, G. Le Duc, S. Roux and O. Tillement, *Small*, 2014, **10**, 1116-1124.

S7. M. Brust, M. Walker, D. Bethell, D.J Schiffrin, R. Whyman, *J. Chem. Soc. Chem. Com*. 1994, **7**, 801-802.

S8. J.A. LaVerne, *Radiat. Res*., 2000, **153**, 487-496.

S9. J. Fulford, P. Bonner, D. T. Goodhead, M. A. Hill and P. O'Neill, *J. Phys. Chem*., 1999, **103**, 11345–11349

S10 J. Meesungnoen, M. Benrahmoune, A. Filali-Mouhim, S. Mankhetkorn and J. P. Jay-Gerin, *Radiat. Res.*, 2001, **155**, 269-278.
